# Personal

**Published:** 1902-12

**Authors:** 


### PERSONAL.

Dr. Edwin Henry Wolcott, of Rochester, has been appointed by Governor Odell a member of the board of visitation for the Gowanda State Homeopathic Hospital, in place of Ogden P. Letchworth, declined.

Dr. William House, U. of B., '95, formerly of Buffalo, has removed from Heppner, Oregon, to Weston, in the same state, where he is engaged in the practice of his profession.

Dr. M. F. Clausius, U. of B., '74, late acting assistant surgeon, U. S. Army, recently stationed at Fort Grant, Arizona, has resigned from the military service, and taken up his residence at Palatine, Illinois, where he will again engage in the private practice of his profession.

Dr. and Mrs. Harvey R. Gaylord have recently returned from Europe and have taken apartments at the Touraine, Delaware Avenue, for the winter. Dr. Gaylord has resumed his professional work and his laboratory investigations.

Dr. J. Hilton Waterman, of New York, has removed from 24 West 50th Street to 50 West 51st Street. Hours: 10 to 11 a.m., and 5 to 6 p.m. Telephone, 6078-38 St.

Dr. and Mrs. Charles A. Ring, have closed their country home, Appleton Hall, and taken apartments at the Castle Inn for the winter.

Hon. Wilson S. Bissell, of Buffalo, has been appointed chancellor of Buffalo University vice Hon. James O. Putnam, resigned. Mr. Bissell has held the office of vice-chancellor for some years past.

Dr. Thomas Hunt Stucky, of Louisville, professor of medicine in the Hospital College of Medicine, Medical Department Central University of Kentucky, recently paid a brief visit to Buffalo.

Dr. Edwin Walker, of Evansville, Indiana, was elected president of the Mississippi Valley Medical Association at its recent annual meeting held in Kansas City.

Dr. Prescott LeBreton, of Buffalo, announces the removal of his office and residence from 122 North Pearl Street to 20 Carlton Street, one door from Main Street.

